# Emerging triboelectric nanogenerators for the prevention and monitoring of inflammation

**DOI:** 10.3389/fimmu.2023.1167301

**Published:** 2023-05-31

**Authors:** En Zhao, Cong Hu, Zhiyuan Zhu

**Affiliations:** ^1^ Chongqing Key Laboratory of Nonlinear Circuits and Intelligent Information Processing, College of Electronic and Information Engineering, Southwest University, Chongqing, China; ^2^ Guangxi Key Laboratory of Automatic Detecting Technology and Instruments, Guilin University of Electronic Technology, Guilin, China

**Keywords:** inflammation, triboelectric nanogenerators, artificial intelligence, prevention, monitoring

## 1 Introduction

A leading thinker on inflammation wrote that “inflammation is associated with almost every major human disease” (1). Inflammation is a defense process of the body against stimuli and a biological response of the immune system to harmful stimuli (2). Inflammation is usually beneficial, but once the inflammatory reaction is out of balance, it will be harmful to the body (3). Inflammation has a great impact on human health, which has been paid more and more attention by researchers. As far as 2021 is concerned, 13905 articles identified “inflammation” as the keyword, and 1284 articles included the word “inflammation” in the title (4). This not only reflects the importance of studying inflammation, but also emphasizes the urgency of inflammation research.

Dysregulated inflammatory reaction can lead to infectious, autoimmune, neurological, cardiovascular, renal and tumor diseases (5–8). Although researchers have put a lot of efforts into biological understanding and drug development, and some interventions have been successful in clinical trial (4), the prevention and monitoring of some inflammation is still a problem. Early monitoring of the prevention and recovery process can effectively reduce the impact of inflammation on people’s health. Although traditional drugs can effectively prevent inflammation, it is always difficult for people to take drugs frequently. Emerging triboelectric nanogenerators (TENG) provides a new prevention and monitoring scheme to address this challenge (9–11). The latest research progress in soft electronics has proposed flexible and stretchable functional sensors (12–14). TENG combines flexible materials and wearable characteristics, and has been successfully applied in the health and medical fields as implantable medical sensors (15–17), biological sensors (18–21), monitoring sensors (22–24), etc. TENG is lightweight, highly flexible and elasticity (25, 26), and can directly contact the skin or organ surface for inflammation prevention and monitoring.

In this paper, the application of the emerging TENG in the prevention and monitoring of inflammation such as cervical spondylitis, lumbar spondylosis, rheumatoid arthritis has been fully discussed. Flexible medical sensors, biosensors and monitoring sensors made of materials with different characteristics have been successfully applied to the prevention and monitoring of some inflammation and diseases. Additionally, this paper also discusses the advantages of TENG combined with artificial intelligence (AI) in disease prevention and monitoring, and the development of TENG in the medical field promoted by AI. At the end of the article, the challenges of the development of TENG and AI in the field of inflammation are analyzed, and its development prospects are prospected.

## 2 TENG for inflammation

Dysregulation of inflammation can seriously affect health. There are many reasons for its formation, including biological factors, physical factors, chemical factors (27), etc. TENG is a powerful technology to convert mechanical energy into electrical energy, which plays a vital role in physiological signal monitoring (28–30), disease prevention and treatment (31), microbial control (32), etc. For example, Wang et al. reported a TENG-based portable spirometer that effectively monitors lung activity (33). The prevention and monitoring of some inflammations (such as cervical spondylitis, lumbar spondylitis, asthma, etc.) through TENG-based equipment contributes to innovation and development in the field of inflammation. Cheng et al. proposed the application of innovative machine learning in inflammation (34), which is of great significance in the application of TENG and AI in inflammation.

### 2.1 Inflammation prevention

**Figure 1:**
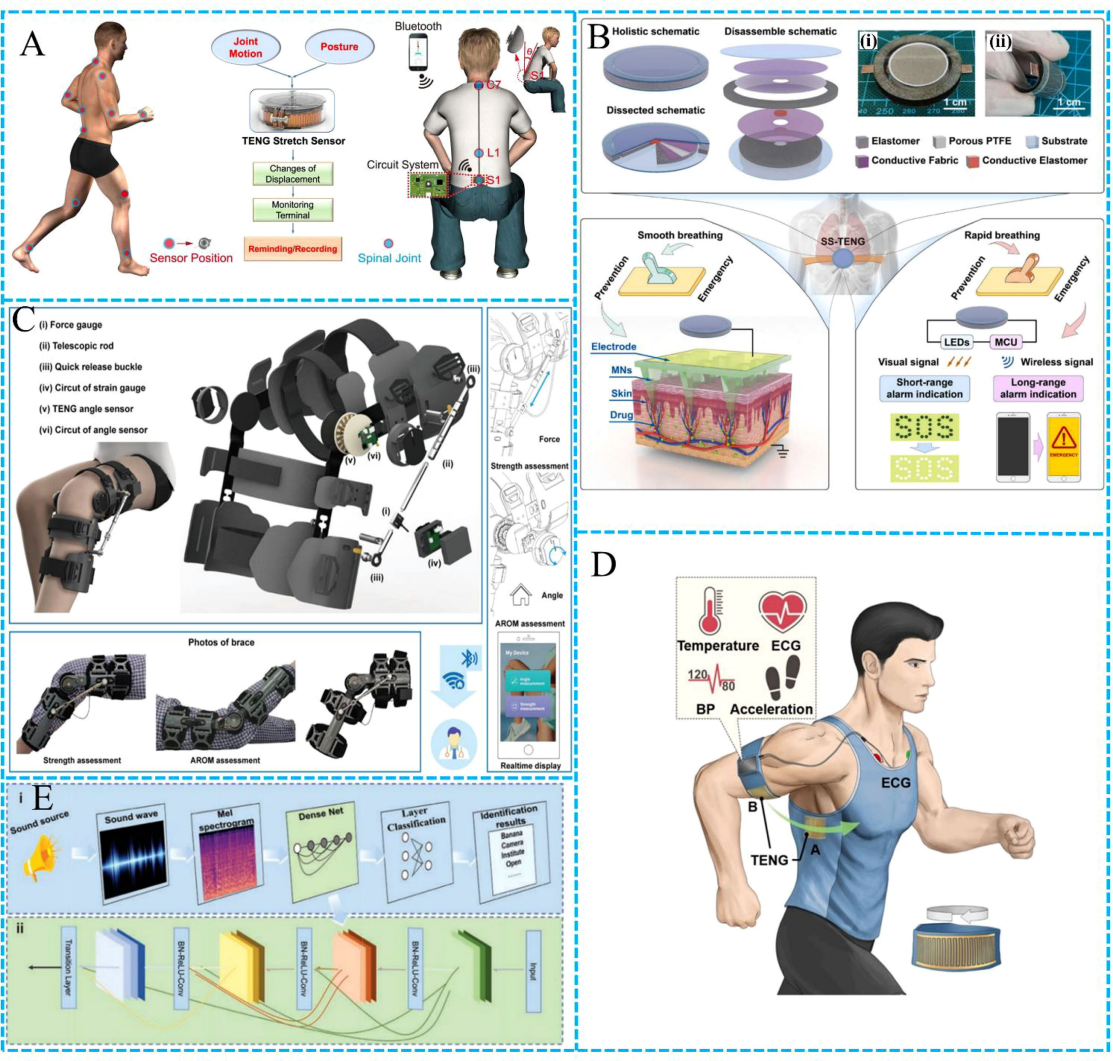
Application of TENG. **(A)** Illustration of the thin, lightweight, and wearable stretch sensor placed around the full body to monitor the joint and spinal motions (35). **(B)** Basic architecture and operation for respiration-mediated self-switched TENG (36). **(C)** Schematic illustration of the rehabilitation brace system (37). **(D)** Schematic diagram of self-powered wireless physiological monitoring system (38). **(E)** The application diagram of ETAS in voice-text conversion (39).

With the improvement of people’s living standards, more and more attention has been paid to the health problems caused by inflammation, and various inflammation prevention, monitoring and treatment technologies have been developed. The development of TENG broadens the boundary for the study of inflammation. Li et al. developed a reel shaped tensile sensing device to reduce the risk of spinal disease (35). The device is a high-precision stretchable sensor that combines retractable badge reel and grating-structured (and the Kapton surface is etched with ions to improve the output performance) (**Figure 1A**). The device is installed at the cervical, lumbar or other joints of the body, and the sensor generates different electrical signals through different stretching or contracting conditions. We can obtain real-time detection signals through the monitoring terminal to judge the state of the spine, which will help reduce the risk of cervical spondylosis, lumbar spondylosis and other spinal diseases caused by abnormal posture.

Yu et al. have developed a self-switched TENG mediated by respiratory motions to provide prevention and emergency functions for asthma (36). The system ingeniously designs an asthma service system with different characteristics of materials such as polyethylene terephthalate (PET) and conductive fabric, which has both prevention and emergency functions and can drive drugs into the body (**Figure 1B**). In the emergency state, the alarm module is driven and send emergency information to the monitoring terminal, so as to effectively prevent and monitor the potential safety hazards caused by asthma.

It is worth noting that these prevention technologies are self-powered, which greatly simplifies the external equipment of traditional prevention technologies and makes prevention more efficient. Therefore, the prevention technology based on TENG has promising application prospects in the field of inflammation.

### 2.2 Inflammation monitoring

Inflammation monitoring is critical. Osteoarthritis is a common inflammation in the elderly, particularly knee osteoarthritis, which not only brings pain and impaired mobility to patients (40, 41), but also may cause social psychological anxiety and depression (42, 43). Luo et al. developed a portable wearable total knee arthroplasty (TKA) patient rehabilitation monitoring and evaluation system based on TENG (37). The system is mainly composed of two sensing modules: force sensor for measuring isometric muscle strength and active angle sensor for detecting joint range of motion (**Figure 1C**). The system visualizes the relationship between torque and force, as well as the range of motion of the patient’s joints, and then transmits the measurement information to the mobile phone for analysis, giving rehabilitation indicators (isometric muscle test score (IMTS)), and quantitatively evaluating TKA rehabilitation.

Yan et al. developed a set of self-powered wireless physiological monitoring system (**Figure 1D**). The TENG and flexible thin-film solar cell are integrated, without external power supply and with high output power (38). The system can effectively monitor inflammation, collect blood pressure, body temperature, movement and other parameters, and transmit the data to the terminal, realizing real-time continuous monitoring of the physical state of the subjects.

The asthma prevention and emergency system based on TENG developed by Yu et al. can not only reduce the incidence rate of asthma patients, but also monitor the status of asthma patients, push drugs for them, and send dangerous signals to the control terminal (36). Lin et al. synthesized 3D dendritic TiO2 nanostructures to prepare ultraviolet radiation (UVR) photodetectors, and this structure can be used for TENG (44) to monitor the intensity of ultraviolet radiation and protect health.

As TENG develops in the field of inflammation, its contribution to inflammation monitoring will increase. The development of TENG can improve our quality of life and even open up new space for telemedicine.

## 3 Application of AI-TENG

Inflammation is linked to several diseases, some of which can damage the eardrum and affect hearing. Jiang et al. reported an ultra-thin eardrum-like triboelectric acoustic sensor (ETAS) that is a boon for people with hearing loss (39). The equipment is assembled by electrospinning technology after silver plating on the nanofiber film, and it is thin and highly sensitive. ETAS is combined with AI algorithm to achieve real-time voice conversion, with a high recognition rate (92.64%), as shown in **Figure 1E**. The system combines the TENG and AI algorithm, so that hearing impaired people have a clear opportunity to listen to the world, and also provides new ideas for inflammation research. Liu et al. also reported a self-powered artificial auditory pathway based on TENG (45), which is composed of a TENG and a field effect synaptic transistor. TENG converts the collected voice signal into electrical signal, and the transistor performs signal conversion and neural morphology operation to simulate the biological acoustic function. The system uses k-nearest neighbors (KNN) algorithm to effectively implement sound detection.

As we all know, the current social development is closely related to AI technology, and AI technology also plays an important role in medical treatment. The deep learning model provides great help for disease prediction, diagnosis and even treatment (46, 47). TENG-based sensors can collect a large amount of data and provide the data basis for AI algorithm. The combination of the two has played a crucial role in medical treatment (48). Therefore, AI-TENG has great research value and application prospect in the field of inflammation.

Although TENG and AI can be helpful for inflammation prevention and monitoring, there are still some limitations in the application of AI technology in TENG. For example, Many AI technologies still lack universal mechanisms, fail to reveal the underlying relationships between structures and materials, and require further exploration between features and predictions in AI models (49–51). Further, for actual prevention and monitoring, there is an urgent need to address specific AI models corresponding to different types of inflammation, and the method to collect and analyze real-time data.

## 4 Discussion

TENG has many advantages, including low cost, high efficiency, softness, wearable, self-powered, and combined with AI algorithm, it has great application prospects in the prevention and monitoring of inflammation, even in the treatment. However, the development of new things always faces many challenges. Here, we divide the challenge into two parts, one is the challenge of AI in TENG: Firstly, most AI technologies cannot reveal the potential relationship between materials, structures and outputs. Secondly, there is a lack of generalized mechanism or understanding of the triboelectric effect. Finally, the over fitting related to AI algorithm, which heavily depends on the quality of input data, is also a challenge. The second is the challenge of AI-TENG in the field of inflammation. First of all, how to analyze the collected data is a problem. Secondly, how to design appropriate algorithms for inflammation research is also a great challenge. Finally, how to evaluate the inflammatory state according to real-time data and AI algorithm. Today, with the rapid development of 5G networks and cloud computing, we have enough opportunities to meet these challenges.

The application of TENG and AI technology to the field of inflammation provides a new direction for inflammation research and new hope for some difficult to solve inflammation. On the one hand, TENG has strong sensing and data collection capabilities, and its structure is variable, so it can design different TENG for different inflammation. On the other hand, AI technology involves many algorithms, such as artificial neural network (ANN), decision tree, linear classifier, etc. We can choose different algorithms for different inflammation to achieve accurate prevention, monitoring and treatment. Additionally, the combination of AI and TENG can not only design and optimize the TENG according to the actual demand, but also optimize the AI algorithm subject to the actual situation. The combination of TENG and AI technology has great application prospects in the field of inflammation. They will make the medical service system more real-time, accurate and diverse.

## Author contributions

EZ and ZZ: conceptualization,writing and revise. ZZ and CH: supervision and funding acquisition. All authors contributed to the article and approved the submitted version.
